# Effects of corn processing index and forage source on performance, blood parameters, and ruminal fermentation of dairy calves

**DOI:** 10.1038/s41598-023-45143-x

**Published:** 2023-10-20

**Authors:** A. Ghasemi, A. Azarfar, H. Omidi-Mirzaei, A. Fadayifar, F. Hashemzadeh, M. H. Ghaffari

**Affiliations:** 1 Department of Animal Science, Faculty of Agriculture, Lorestan University, PO Box 465, Khorramabad, Iran; 2 Animal Science Research Department, Isfahan Agricultural and Natural Resources Research and Education Center, AREEO, PO Box 81785-199, Isfahan, Iran; 3 Department of Animal Science, College of Agriculture, Isfahan University of Technology, Isfahan, 84156-83111 Iran; 4 Institute of Animal Science, University of Bonn, 53111 Bonn, Germany

**Keywords:** Developmental biology, Physiology

## Abstract

The objective of this study was to investigate the effects of corn processing index (CPI)—particularly at 70% and 85%—in starter feed in combination with the provision of forage, either alfalfa hay (AH) or wheat straw (WS), on feed intake, growth performance, rumen pH, and blood metabolites of dairy calves. Forty-eight male Holstein calves (43.0 ± 1.5 kg body weight) were randomly assigned (n = 12 calves per treatment) to one of four dietary treatments: (1) a textured starter diet containing 70% CPI and AH (70CPIAH), (2) a textured starter feed containing 70% CPI combined with WS (70CPIWS), (3) a textured starter feed containing 85% CPI and AH (85CPIAH), (4) a textured starter feed containing 85% CPI combined with WS (85CPIWS). Intake of starter feed (TMR) and milk was individually monitored and recorded daily, while body weight was measured weekly. On days 30 and 65, blood samples were collected from all calves 3 h after morning feeding. No interaction was detected between the CPI of starter feed diets and forage provision for starter intake, dry matter intake (DMI), metabolizable energy intake (MEI), feed efficiency (FE), average daily gain (ADG), and body weight (BW). The preweaning and overall DMI, preweaning, postweaning and overall FE and ADG, preweaning and overall starter intake, preweaning and overall ME intake, and postweaning and overall BW were greater for calves fed 85CPI than those fed 70CPI starter diets. Postweaning and overall ADG and postweaning FE were greater in calves fed WS than those fed AH. Body length and hip width were greater for calves offered 85CPI than in fed 70CPI. Wheat straw supplementation increased rumen pH at day 65 in calves fed 70CPI, but not in those fed 85CPI. No interaction was found between the CPI in the starter feed diet and the forage source for starter intake and DMI, MEI, FE, and BW. The results showed that including corn with 85% processing index in dairy calf starters improved their growth performance.

## Introduction

To achieve optimal early growth in dairy calves, it is essential to develop an effective calf rearing program. One pivotal factor in this regard is the inclusion of grains, primarily starch sources, in calf starter feed diets. Indeed, the structural properties of the endosperm of these cereal grains play a critical role in determining the site and rate of starch digestion in ruminants^1^. In addition, the selection of grains processing techniques, when matched with the physical properties of the starter feed, has a major impact on the transition from a pre-ruminant status to a fully developed ruminant status^2,3^.

Recent studies emphasize the significant influence of grain and starter feed processing on rumen fermentation dynamics and nutrient assimilation in dairy calves^4–6^. In particular, steam-flaked corn has become the predominant component in calf starter diets. This processing method is thought to enhance starch gelatinization^7^, thereby optimizing both ruminal and post-ruminal starch digestion^8^.

However, the use of steam-flaked corn in the diets of young calves has produced inconsistent results. Lesmeister and Heinrichs^4^ observed a reduction in ADG, starter feed intake, and FE with steam-flaked corn compared to whole or dry-rolled corn grain. In contrast, Mojahedi et al.^5^ found negligible differences in performance or bone growth between calves fed steam-flaked corn and those fed cracked corn. Interestingly, their research showed that calves’ growth performance was enhanced when they were fed forage in addition to steam-flaked corn. This was particularly evident when contrasted with a diet of cracked corn, both in the preweaning periods^5^.

It is plausible that excessive processing of grain, severe gelatinization of starch, or an abundance of fine particles could lead to accelerated production of rumen acids. Such an environment could potentially disrupt rumen fermentation processes and subsequently affect growth performance of dairy calves^4,9^. There are data suggesting that feeding ground grain to calves may improve their performance. This improvement is attributed to stabilization of rumen pH, which promotes a more uniform fermentation environment^2,6,10^.

The Processing Index (PI) serves as a metric that quantifies the extent of grain processing^11^. A decrease in grain density signifies a more thorough rolling process, thus resulting in a reduced PI^12^. Moreover, research indicates a rise in starch digestion, both in the rumen and total tract, when corn flake density diminishes^13^. Yang et al.^14^, in their exploration of the implications of varying PI levels of barley, unveiled that an optimal PI of 64% maximizes milk yield in dairy cows without adversely affecting the milk fat percentage.

Focusing on proper corn processing ensures that a substantial proportion of starch becomes accessible to rumen microorganisms and enzymes. This, in turn, amplifies production performance and FE in dairy calves^16^. Nevertheless, there exists an inherent risk with calves ingesting large quantities of swiftly fermentable carbohydrates might suffer from digestive disorders like ruminal acidosis. This could adversely affect both their health and performance^12,17^. A judicious inclusion of roughage can circumvent such digestive complications, ensuring that growth performance and feed efficiency remain unaffected. For instance, Ran et al.^12^ established that elevating undigested NDF (uNDF) in the diet enhances rumen pH in heifers fed with highly processed barley.

Nevertheless, there are few studies on the interaction between variations in CPI of the starter and type of forage and their combined effects on growth performance of dairy calves fed textured starter feed. Therefore, the current study examined how rumen fermentability, which is affected by the degree of corn processing, affects production performance of dairy calves as a function of the type of forage in the starter diet. Specifically, we will examine the effects of processing corn at two indices (70% and 85%) in conjunction with two forage types (alfalfa hay (AH) and wheat straw (WS)).

## Materials and methods

### Animals, management, and treatments

The current study was conducted between September 19 and December 12, 2019, at a local dairy farm (Khoramdareh Agri. Animal Production Co., Zanjan, Iran). Ethical approval for all procedures involving animals was obtained from the Animal Care and Use Committee of Lorestan University (LUT, Iran; LACUC #2022/D06) before the start of the study. The study complies with ARRIVE guidelines for reporting in vivo experiments, and all methods were performed following the relevant guidelines and regulations. Experimental research and field studies on plants (either cultivated or wild), including the collection of plant material, must comply with relevant institutional, national, and international guidelines and legislation. Forty-eight male Holstein calves (43 ± 1.5 kg body weight) were randomly assigned (n = 12 calves per treatment) to a 2 × 2 factorial arrangement of treatments with factors of corn processing index (CPI) of starter feed (70 vs. 85%) and forage source (AH or WS). Calves were separated from their mothers immediately after birth, weighed, and housed in individual pens (1.2 × 2.5 m) that were bedded with sand and renewed every 2–3 days. Calves obtained at the birth, received 3 L of colostrum at each of the first two feedings (i.e., within 2 h of life and 12 h after the first feeding) according to the farm’s protocol. Calves were only included in the experiment if they successfully achieved passive immunity, indicated by a serum IgG concentration of ≥ 25 g/L. Thereafter, colostrum feeding (4 L/day) was continued until the end of the third day. Calves received 5 L/day of whole milk in steel buckets twice daily at 0900 and 1800 from day 4 to day 50 of the study, followed by feeding 2.5 L/day of milk until day 56 of the study when they were weaned^6^. Calves were assigned to one of four feeding treatments: (1) a textured starter feed containing 70% CPI and AH (70CPIAH), (2) a textured starter feed containing 70% CPI and WS (70CPIWS), (3) a textured starter feed containing 85% CPI and AH (85CPIAH), (4) a textured starter feed containing 85% CPI and WS (85CPIWS). The textured starter feed contained steam-flaked corn in combination with other pelleted ingredients, with the exception of forage sources. Both starters had the same ingredients and nutrient composition, but differed in their CPI. A single batch of corn grain was used to produce corn grain with different processing index. The corn grains were sieved and steamed at 95 °C for 30 min in a stainless-steel chamber. Then the steamed grain was passed through a roller mill. The roller tension and spacing were adjusted to achieve a processing index of 85 and 70, respectively. Processing index (PI) = weight per unit volume after processing expressed as a proportion of the weight per unit volume before processing^11^. In this experiment, weight per unit volume before processing corn was 585 g, weight per unit volume after processing corn for 70 PI was 409 g, and weight per unit volume after processing corn for 85 PI was 497 g. Calves were weaned on day 56 (preweaning period = days 3–56) and remained in the study until day 70 (postweaning period = days 57–70). From day four of life, all calves had free access to fresh water and starter feed throughout the study. The feed offered was adjusted daily to achieve a proportion of 5 to 10% orts (i.e., the portion of the starter that was not consumed within 24 h); orts were collected and weighed at 0900 h daily. The starter diet offered contained 5% chopped AH (geometric mean particle size 2.5 ± 0.63 mm) or WS (geometric mean particle size 2.8 ± 0.5 mm) as total mixed ration (TMR) throughout the study. Forage plants were chopped using a stationary forage chopper (Isfahan Machinery co, Isfahan, Iran) with a theoretical chop length of 4 mm. The starter feed was formulated in accordance with NRC (2001)^18^. The chemical composition of the main ingredients of the starter feed is shown in Table 1. The ingredients and nutrient composition of the starter feed are listed in Table 2.

**Table 1:** Mean (± SD) chemical composition (% of DM) of corn, soybean meal, alfalfa hay, and wheat straw (%).

Item	Corn	Soybean meal	Alfalfa hay	Wheat straw
DM	89.3 ± 1.0	88.7 ± 1.4	90.6 ± 1.4	92.00 ± 1.2
OM	97.6 ± 0.1	94.4 ± 0.3	89.7 ± 0.1	90.4 ± 0.6
CP	9.2 ± 0.4	47.8 ± 0.3	14.3 ± 0.7	4.4 ± 0.3
EE	4.3 ± 0.2	1.6 ± 0.6	2.62 ± 0.7	1.21 ± 0.3
NDF	9.8 ± 0.5	15.4 ± 0.5	45.1 ± 1.2	73.1 ± 0.2

*DM* dry matter, *OM* organic matter, *CP* crude protein, *EE* ether extract, *NDF* neutral detergent fiber.

**Table 2:** Ingredients, chemical composition, and particle size distribution of experimental diets.

Item	70CPI-AH	85CPI-AH	Treatments	
			70CPI-WS	85CPI-WS
Ingredients (% of DM)				
Alfalfa hay	5.00	5.00	–	–
Wheat straw	–	–	5.00	5.00
Steam-flaked corn	55.00	55.00	54.60	54.60
Soybean meal	23.30	23.30	23.40	23.40
Pishgam permix	15.00	15.00	15.20	15.20
Salt	0.50	0.50	0.50	0.50
Calcium carbonate	1.20	1.20	1.30	1.30
Chemical composition, (% of DM)				
DM	88.00	88.00	89.00	89.00
CP	20.90	20.90	20.40	20.40
NDF	16.10	16.10	16.40	16.40
ADF	11.00	11.00	11.60	11.60
ME	3.16	3.16	3.14	3.14
NEg	1.49	1.49	1.47	1.47
NFC^2^	58.00	58.00	57.00	57.00
Ca^3^	0.73	0.73	0.72	0.72
P^3^	0.50	0.50	0.49	0.49
g/kg of particles retained on the sieve				
4.75 mm	30.00	40.20	32.00	43.40
2.36 mm	60.00	55.00	58.00	53.10
1.18 mm	4.00	2.30	3.50	2.00
0.6 mm	3.25	1.60	3.00	1.30
0.3 mm	2.70	0.90	2.49	0.20
0.15 mm	0.05	0.00	0.01	0.00
< 0.15 mm	0.00	0.00	0.00	0.00
GMPL, mm	2.56	2.95	2.77	3.08

*CPI* corn processing index, *AH* alfalfa hay, *WS* wheat straw, *GMPL* geometric mean particle length; calculated as described by the American Society of Agricultural Engineers (1983), *ME* metabolizable energy (Mcal/kg), *NEg* net energy for gain (Mcal/kg), *CP* crude protein, *NDF* neutral detergent fiber, *NFC* non-fiber carbohydrate.

^1^Contained per kilogram of supplement (unless noted): DM = 93%, CP = 29%, Fat = 6.5, ME = 2.31 (Mcal), NEg = 0.85 Mcal, NDF = 17.5%, Ca = 0.65%, P = 0.77%, NFC = 29%, Mg = 2%, K = 0.99%, Na = 1.6%, Cl (mg/kg) = 0.1, Co (mg/kg) = 23, Mn (mg/kg) = 43, Se (mg/kg) = 0.1, Zn (mg/kg) = 43, Vit A (IU) = 12,000, Vit D3 (IU) = 5000, Vit E (IU) = 100.

^2^Calculated as DM − (NDF + CP + ether extract + ash) (NRC, 2001).

^3^Estimated using the NRC (2001) model.

### Laboratory analysis

Intake of starter feed (TMR) and milk was individually monitored and recorded daily, while body weight was measured weekly with an electronic scale throughout the experimental period. Simultaneously, feed and orts samples were collected weekly throughout the study and subsequently stored at − 20 °C for chemical analysis. These collected samples were assembled monthly, dried at 55 °C for 48 h, and then pulverized using a Wiley mill (Ogaw Seiki Co., Ltd., Tokyo, Japan) to ensure that they fell through a 1-mm sieve before chemical analyzes were performed. The above chemical assays were performed to quantify dry matter (DM, method 934.01)^19^, crude protein (CP, method 988.05)^19^, lipids (method 920.39)^19^, and neutral detergent fiber (NDF) content using heat-stable α-amylase (100 μL/0.5 g of sample) and sodium sulfite to quantify^20^. Throughout the experimental period, a total of ten milk samples were collected and subsequently analyzed for components such as fat, CP, lactose, and total solids content using Milkoscan (Foss Electric, Hillerød, Denmark). Total DMI was calculated by combining total solids derived from milk and starter feed. Average daily gain (kg/day) and FE (ADG/total DMI) were calculated. At weaning age (day 56) and at the end of the study (day 70), the physical parameters of the calves, including body length, withers height, heart girth, hip height, and hip width, were measured using the method described in Omidi-Mirzaei et al.^6^.

Before the experiment, AH and WS were chopped (Golchin Trasher Hay Co., Isfahan, Iran). Throughout the experiment, eight starter samples were collected from each treatment and used for particle size distribution. Particle size distribution of four experimental forages and AH was measured by dry sieving using an automatic vibrating sieve shaker (Model 120; Techno Khak, Khavaran, Tehran, Iran) with sieves of 4.75, 2.36, 1.18, 0.6, 0.3, and 0.15 mm in diameter (Table 2). Exactly 100 g of the sample was placed on the top sieve in duplicate and the sieve stack was shaken until the distribution of the materials did not change (about 10 min). Geometric mean particle size was calculated as described by the American Society of Agricultural Engineers^21^. On days 35 and 65, rumen fluid was collected 3 h after feeding using a vacuum pump and a stomach tube. The first 50 mL was discarded to minimize the risk of saliva contamination. The pH of the sample was determined using a pre-calibrated pH meter (HI 8318; Hanna Instruments, Cluj-Napoca, Romania).

On day 30 and 65, blood samples were collected from all calves 3 h after morning feeding. Vacuum-assisted tubes containing K2 EDTA (from Becton Dickinson Vacutainer Systems, Franklin Lakes, NJ, USA) were used for blood collection from the jugular vein and immediately placed on ice. The collected blood samples were immediately centrifuged at a force of 2850×*g* and kept at 4 °C for 20 min. Subsequently, a volume of 1.5 mL of plasma was divided into 2 mL cryotubes and stored at − 20 °C in preparation for further analysis. Blood metabolites were analyzed by a spectrophotometric method using the UNICCO 2100 instrument (supplied by Zistchemi Co., Tehran, Iran). Commercially available test kits from Pars Azmoon Company (Tehran, Iran) with the following catalog numbers were used: Glucose (1-500-017), Triglycerides (TG; 1-500-032), and Very Low-Density Lipoproteins (VLDL, 1-600-043). The analysis was performed strictly according to the manufacturer’s instructions. Plasma BHB concentration was determined with an autoanalyzer and kit from Randox Laboratories Ltd (Ardmore, United Kingdom). The intra- and inter-assay values CV for BHB were 6.9 and 8.2%, respectively. The intra- and inter-assay values CV were 3.4 and 2.9%, respectively, for glucose.

### Statistical analyses

Following previously published values^22,23^, a daily standard deviation of 100 g ADG was assumed, and a difference of 65–75 g per day was considered meaningful. A power test analysis was performed with α = 0.05 and power (1 − β) = 0.80, resulting in an expected sample size of 12 calves per treatment for growth performance. This parameter can be used to most accurately determine power. Data were analyzed using a completely randomized experimental design with a 2 × 2 factorial arrangement of forage source (AH and WS) and CPI of starter treatments and analyzed using the MIXED procedure of SAS with analysis of variance (ANOVA). Time served as a repeated measure of starter feed intake, total DMI, ADG, FE, ME intake, skeletal growth, rumen pH, and selected blood metabolites, with the individual calf as the experimental unit. The model included fixed effects of forage source, CPI of starter, time, and their interactions, with the calf included as a random effect. The main effects of forage source, CPI of starter, and interactions were tested using ANOVA.

The statistical model employed is represented by: *Y*_*ijkl*_ = *µ* + *P*_*j*_ + *F*_*k*_ + *T*_*l*_ + (*PT*)_*jl*_ + (*FT*)_*kl*_ + (*PF*)_*jk*_ + (*PFT*)_*jkl*_ + *A*_*i*_ + *β*(*Xi − X*) + ε_*ijkl*_. Where *Y*_*ijkl*_: dependent variable; *µ*: Overall mean; *P*_*j*_: Fixed effect of the *j*th CPI of starter; *F*_*k*_: Fixed effect of the *k*th forage source; T_*l*_: Fixed effect of the *l*th period; (*PT*)_*jl*_: interaction effect between the *j*th CPI of starter and the *l*th period; (FT)_*kl*_: Interaction effect between the *k*th forage source and the *l*th period; (*PF*)_*jk*_: Interaction between the *j*th CPI of starter and the *k*th forage source; (*FPT*)_*jkl*_*:* Three-way effect among the *j*th CPI of starter, the *k*^*th*^ forage source, and the *l*th period; *A*_*i*_: Random effect of the *i*th calf; *β*(*Xi − *$X$): Covariate variable where *Xi* is the individual observation and *X* is the overall mean of the covariate. For body weight (BW) and skeletal growth, initial values were considered as covariates. ε_*ijkl*_: Random residual error which is assumed to be normally distributed with mean 0 and variance σ^2^. A type 1 autoregressive covariance structure was selected as the best fit based on the Bayesian information criterion after testing three variance–covariance structures (type 1 autoregressive, compound symmetry, and Toeplitz). Residuals were tested for normality using the Shapiro–Wilk statistic and the UNIVARIATE procedure in SAS, as well as the Levene’s test for homogeneity of variance and quantile–quantile plots for visual assessment. Data that did not meet the assumptions for the normality of the residuals were log-transformed (base 10) including rumen pH and blood glucose concentrations. As a result of the log transformation, the distribution of the data was retested and confirmed to be normally distributed. In the analysis of weaning and final BW (days 56 and 70), the initial BW was used as a covariate. A Turkey–Kramer adjustment was applied to account for multiple comparisons. The significance threshold was set at *P* ≤ 0.05, and trends were explained at 0.05 < *P* ≤ 0.10.

## Results

### Intake and growth performance

**Figure 1:**
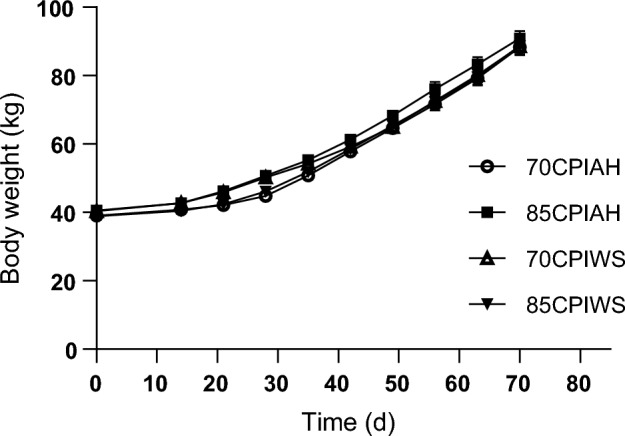
Body weight in calves receiving different experimental diets. Treatments were (1) calves fed a textured starter diet containing 70% corn processing index and alfalfa hay (70CPIAH), (2) calves fed a textured starter diet containing 70% corn processing index and wheat straw (70CPIWS), (3) calves fed a textured starter diet containing 85% corn processing index and alfalfa hay (85CPIAH), (4) calves fed a textured starter diet containing 85% corn processing index and wheat straw (85CPIWS). Data are presented as means ± SEM.

**Figure 2:**
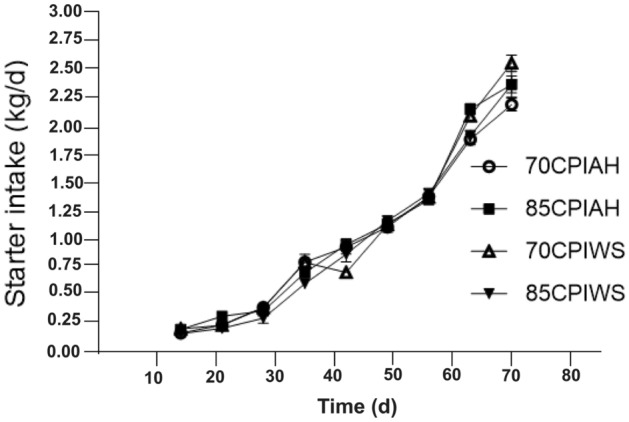
Starter feed intake in calves receiving different experimental diets. Treatments were (1) calves fed a textured starter diet containing 70% corn processing index and alfalfa hay (70CPIAH), (2) calves fed a textured starter diet containing 70% corn processing index and wheat straw (70CPIWS), (3) calves fed a textured starter diet containing 85% corn processing index and alfalfa hay (85CPIAH), (4) calves fed a textured starter diet containing 85% corn processing index and wheat straw (85CPIWS). Data are presented as means ± SEM.

**Figure 3:**
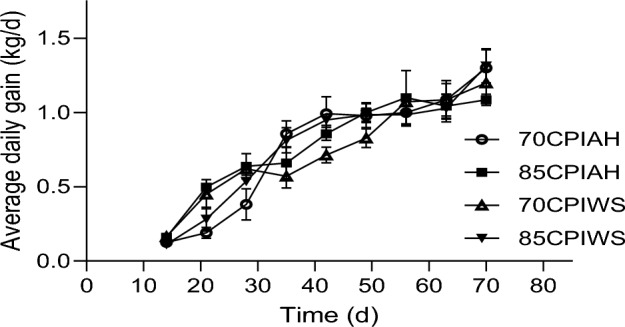
Average daily gain in calves receiving different experimental diets. Treatments were (1) calves fed a textured starter diet containing 70% corn processing index and alfalfa hay (70CPIAH), (2) calves fed a textured starter diet containing 70% corn processing index and wheat straw (70CPIWS), (3) calves fed a textured starter diet containing 85% corn processing index and alfalfa hay (85CPIAH), (4) calves fed a textured starter diet containing 85% corn processing index and wheat straw (85CPIWS). Data are presented as means ± SEM.

**Figure 4:**
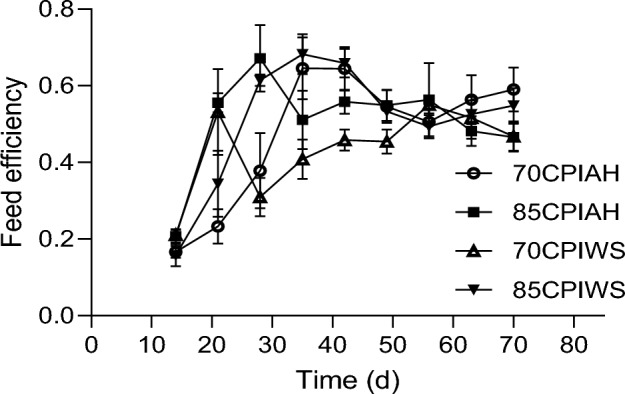
Feed efficiency in calves receiving different experimental diets. Treatments were (1) calves fed a textured starter diet containing 70% corn processing index and alfalfa hay (70CPIAH), (2) calves fed a textured starter diet containing 70% corn processing index and wheat straw (70CPIWS), (3) calves fed a textured starter diet containing 85% corn processing index and alfalfa hay (85CPIAH), (4) calves fed a textured starter diet containing 85% corn processing index and wheat straw (85CPIWS). Data are presented as means ± SEM.

Body weight (BW; Fig. 1), starter feed intake (Fig. 2), total DMI, and ME intake, ADG (Fig. 3), FE (Fig. 4), and data are reported in Table 3. No interaction was found between the CPI of starter diets and forage source for starter intake and DMI. The preweaning (1.33 vs. 1.09 kg/day; *P* = 0.01), and overall DM intake (1.62 vs. 1.40 kg/day; *P* = 0.02) were greater for calves fed 85CPI starter diets than those fed 70CPI starter diets. The preweaning (0.79 vs. 0.43 kg/day; *P* = 0.02), and overall starter feed intake (0.97 vs. 0.81 kg/day; *P* = 0.01) were greater for calves fed 85CPI starter diets compared with calves fed 70CPI starter diets. However, forage sources did not affect DMI and starter intake in the 70CPI and 85CPI starter diets.

**Table 3:** Effects of corn processing index of starter diets (70 versus 85) and forage source (alfalfa hay and wheat straw) on starter intake, DMI, MEI, ADG, and FE of dairy calves (n = 12 calves per treatment).

Item	70CPI		85CPI		SEM	*P*-value^1^			Time (T)	P × T	F × T	P × F × T
	AH	WS	AH	WS		P	F	P × F				
Total DMI, kg/day												
Preweaning (day 3 to 56)	1.12	1.07	1.35	1.32	0.03	0.01	0.56	0.74	< 0.01	0.36	0.36	0.95
Overall (day 3 to 70)	1.39	1.40	1.60	1.64	0.05	0.02	0.61	0.81	< 0.01	0.43	0.27	0.87
Starter intake, kg/day												
Preweaning (day 3 to 56)	0.41	0.46	0.72	0.87	0.03	0.02	0.41	0.84	< 0.01	0.01	0.48	0.76
Postweaning (day 57 to 70)	1.95	2.04	2.11	2.19	0.11	0.78	0.34	0.92	< 0.01	0.76	0.69	0.56
Overall (day 3 to 70)	0.82	0.80	0.95	1.00	0.04	0.01	0.79	0.89	< 0.01	0.01	0.57	0.65
ME intake, Mcal/day												
Preweaning (day 3 to 56)	4.31	4.18	5.10	4.98	0.10	0.01	0.19	0.90	< 0.01	0.01	0.56	0.54
Postweaning (day 57 to 70)	6.55	6.84	7.00	7.02	0.30	0.79	0.31	0.86	< 0.01	0.78	0.45	0.48
Overall (day 3 to 70)	5.40	5.49	6.01	5.85	0.15	0.04	0.87	0.86	< 0.01	0.01	0.09	0.78
ADG, kg/day												
Preweaning (day 3 to 56)	0.46	0.44	0.52	0.55	0.02	0.01	0.69	0.41	< 0.01	0.01	0.47	0.18
Postweaning (day 57 to 70)	0.79	0.80	0.89	0.95	0.02	0.01	0.18	0.38	< 0.01	0.03	0.43	0.37
Overall (day 3 to 70)	0.62	0.63	0.73	0.76	0.02	0.01	0.23	0.69	< 0.01	0.01	0.04	0.16
Feed efficiency^2^												
Preweaning (day 3 to 56)	0.41	0.42	0.45	0.47	0.03	0.03	0.58	0.49	< 0.01	0.14	0.08	0.23
Postweaning (day 57 to 70)	0.39	0.41	0.42	0.43	0.01	0.01	0.03	0.45	< 0.01	0.01	0.15	0.17
Overall (day 3 to 70)	0.40	0.41	0.43	0.45	0.02	0.02	0.19	0.78	< 0.01	0.03	0.01	0.33
BW, kg												
Initial (day 3)	42.31	42.55	42.12	42.10	1.19	0.65	0.83	0.79				
Weaning (day 56)	67.49	67.01	71.04	72.49	1.28	0.01	0.44	0.59				
Final (day 70)	78.70	78.13	83.44	85.45	1.25	0.01	0.47	0.82				

^1^Statistical comparisons: *P* corn processing index (CPI), *F* forage source, *T* period, *P* × *F* processing index × forage source, *P* × *T* processing index × period, *F* × *T* forage source × period, *P* × *F* × *T* processing index × forage source × period.

^2^Feed efficiency = kg of BW gain/kg of total DMI.

No significant interaction was found between CPI the starter diet and the forage source for ME intake. The preweaning (5.04 vs. 4.24 Mcal/day; P = 0.01) and overall ME intake (5.93 vs. 5.44 Mcal/day; P = 0.04) were greater in calves fed 85CPI starter diets than in calves fed 70CPI starter diets. The pre- weaning (4.70 vs. 4.58 Mcal/day; P = 0.19), post-weaning (6.77 vs. 6.93 Mcal/day; P = 0.31), and overall ME intake (5.70 vs. 5.67 Mcal/day; P = 0.87) were similar in AH-fed calves compared with WS-fed calves. No interaction was observed between the CPI of starter diets and forage source for ADG during the preweaning (*P* = 0.41), postweaning (*P* = 0.38), and overall (*P* = 0.69) periods. In the current study, the preweaning (*P* = 0.01), postweaning (*P* = 0.01), and overall ADG (*P* = 0.01) were greater in calves fed 85CPI than those fed 70CPI. The preweaning (*P* = 0.69), postweaning (*P* = 0.18), and overall ADG (*P* = 0.23) were similar for AH and WS-supplemented calves.

No significant interaction was found between the CPI of starter diets and the forage sources for FE. Preweaning (P = 0.03), postweaning (P = 0.01), and overall FE (P = 0.02) values were higher in calves fed 85CPI than in those fed 70CPI. The FE was greater after weaning (P = 0.03) in calves fed WS than in calves fed AH. The BW of the calves were similar at the beginning of the experiment. No significant interaction was observed between CPI and forage source for weaning and final weight. Calves fed 85CPI starter diets had higher weaning and final weights (P = 0.01; Table 3) than calves fed 70CPI starter diets. Calves fed 85CPI starter diets had greater weaning and average body length (P < 0.05). There was an interaction between CPI and time for hip width, with calves offered the 85CPI starter feed having greater final hip width than those offered the 70CPI starter feed (P < 0.05). The effects of the CPI and the forage sources were similar (P ≥ 0.05) for weaning and final withers height, heart girth, and hip height (P > 0.05, Table 4).

**Table 4:** Effects of corn processing index of starter diets (70 versus 85) and forage source (alfalfa hay and wheat straw) on body measurements of dairy calves (n = 12 calves per treatment).

Item (cm)	70 CPI		85 CPI		SEM	*P*-value^1^			Time (T)	P × T	F × T	P × F × T
	Alfalfa hay	Wheat straw	Alfalfa hay	Wheat straw		P	F	P × F				
Body length												
Weaning	42.1	43.0	46.4	45.9	1.28	< 0.01	0.84	0.61				
Final	48.2	48.9	50.1	50.5	1.26	0.18	0.66	0.90				
Overall	45.2	46.0	48.2	48.2	0.89	< 0.01	0.66	0.65	< 0.01	0.30	0.85	0.78
Wither height												
Weaning	84.7	84.1	85.3	85.6	0.82	0.19	0.87	0.61				
Final	92.5	92.1	93.8	93.9	0.92	0.10	0.88	0.75				
Overall	88.7	88.0	89.1	89.1	0.61	0.23	0.59	0.64	< 0.01	0.61	0.76	0.79
Heart girth												
Weaning	97.0	97.3	97.6	98.0	0.82	0.43	0.67	0.92				
Final	103.3	102.1	103.4	104.1	1.18	0.38	0.83	0.41				
Overall	100.1	99.7	100.5	101.1	0.72	0.23	0.94	0.48	< 0.01	0.78	0.68	0.52
Hip width												
Weaning	18.3	17.9	17.9	18.2	0.29	0.78	0.76	0.23				
Final	19.3	19.0	19.8	20.0	0.30	0.01	0.99	0.43				
Overall	18.8	18.5	18.9	19.0	0.21	0.10	0.82	0.16	0.01	0.04	0.83	0.77
Hip height												
Weaning	85.1	84.6	85.2	85.7	0.94	0.54	0.95	0.55				
Final	92.6	92.0	92.9	92.5	0.87	0.62	0.56	0.88				
Overall	88.8	88.3	89.5	89.8	0.65	0.10	0.88	0.54	< 0.01	0.43	0.94	0.88

^1^Statistical comparisons: *P* corn processing index (CPI), *F* forage source, *T* period, *P* × *F* processing index × forage source, *P* × *T* processing index × period, *F* × *T* forage source × period, *P* × *F* × *T* processing index × forage source × period.

### Blood metabolites and rumen pH

Data on plasma concentrations of glucose, triglycerides, BHB and ruminal pH are reported in Table 5. Rumen pH was affected by both CPI and forage sources (P < 0.05). Calves offered the starter feed containing 85CPI or WS had higher rumen pH at day 35 than calves fed the feed containing 70CPI or AH. In addition, there was an interaction between CPI and forage sources for final and overall rumen pH, suggesting that supplementation of WS increased rumen pH in calves fed a starter diet containing 70CPI, but this response was not observed when calves were fed a starter diet containing 85CPI. Calves fed a starter feed containing 85CPI had greater blood glucose (P < 0.01) and BHB (P = 0.05) concentrations throughout the study period. The CPI, forage source, and their interaction did not affect plasma concentrations of triglycerides (days 30 and 65 of the study) and BHB (day 65).

**Table 5:** Effects of corn processing index of starter diets (70 versus 85) and forage source (alfalfa hay and wheat straw) on ruminal pH, glucose, triglycerides, and BHB of dairy calves (n = 12 calves per treatment).

Item	70 CPI		85 CPI		SEM	*P*-value^1^			Time (T)	P × T	F × T	P × F × T
	Alfalfa hay	Wheat straw	Alfalfa hay	Wheat straw		P	F	P × F				
Ruminal pH												
Day 30	5.65	5.75	5.89	5.95	0.04	< 0.01	0.03	0.44				
Day 65	5.15	5.42	5.75	5.84	0.02	< 0.01	< 0.01	< 0.01				
Overall	5.40	5.58	5.82	5.89	0.02	< 0.01	< 0.01	< 0.01	< 0.01	< 0.01	< 0.01	0.12
Glucose, mg/dL												
Day 30	99.10	98.40	114.5	118.5	6.43	0.01	0.80	0.71				
Day 65	67.10	68.70	75.50	78.60	2.89	< 0.01	0.42	0.80				
Overall	83.10	83.50	95.00	98.60	3.52	< 0.01	0.57	0.66	< 0.01	0.23	0.91	0.82
Triglycerides, mg/dL												
Day 30	20.40	19.20	18.90	20.00	2.64	0.88	0.98	0.67				
Day 65	33.10	32.90	33.20	34.50	4.6	0.85	0.90	0.88				
Overall	26.70	26.10	26.00	27.30	2.67	0.92	0.91	0.73	< 0.01	0.82	0.91	0.93
BHB, mmol/L												
Day 30	0.16	0.14	0.18	0.21	0.02	0.02	0.78	0.18				
Day 65	0.34	0.32	0.32	0.35	0.02	0.70	0.78	0.31				
Overall	0.25	0.23	0.25	0.28	0.01	0.05	0.69	0.10	< 0.01	0.21	0.96	0.89

^1^Statistical comparisons: *P* corn processing index (CPI), *F* forage source, *T* period, *P* × *F* processing index × forage source, *P* × *T* processing index × period, *F* × *T* forage source × period, *P* × *F* × *T* processing index × forage source × period.

## Discussion

### Intake and growth performance

In this study, the greater total DMI and intake of starter feed in calves fed 85CPI compared with calves fed 70CPI, as reflected by higher ADG, was likely due to lower rumen pH and higher passage rate. It has been reported that with similar starter feed ingredients, processing grain into a significant proportion of fine particles can disrupt rumen epithelial health and reduce feed intake and ADG in dairy calves^7,24^. More importantly, they found that manufacturing processes did not affect calf performance when starter diets contained similar ingredients and nutrient levels, unless the diets contained a significant proportion of large particles that affected starter feed intake and thus ADG^7^. Presumably, the 70CPI starter diet contained a high proportion of fine particles that reduced calf feed intake. In our experiment, feeding calves 85CPI increased DMI and ruminal pH compared with calves fed 70CPI, suggesting that the 85CPI diet contained less readily rumen-degradable starch than the 70CPI diet. In support of our speculation, Zinn^25^ reported that reducing the density of steam-flaked corn in the diets of feedlot cattle from 0.42 to 0.30 kg/L lowered rumen pH and predisposed them to rumen acidosis. In the current study, forage source had no effect on DMI and starter feed intake in the 70CPI starter diets. Recently^12^, it was found that in heifers fed a high grain diet, a decrease in PI decreased rumination activity and increased digestibility of OM, starch, and CP throughout the tract without affecting rumen pH and fermentation parameters. It was also found that in heifers, a PI of 75% was optimal for feed digestibility, as further reduction of PI to 65% did not further improve digestibility^12^. This may indicate that in the present study the extent of corn processing indicated as PI had a more profound effect on calves’ performance than does forage type. However, in some studies^2,6,26^, provision of forage in calves’ starter diet enhanced starter feed intake and ADG compared with calves fed steam-flaked corn in a textured diet without forage provision. Studies investigating the impact of forage provision to dairy calves on starter intake have yielded inconsistent results owing to factors such as forage levels, forage sources, and the physical form of starter feeds used^10^.

In our study, 70CPI starter diets resulted in lower intake and weight gain compared with 85CPI starter diets, regardless of feed type. The greater pre and postweaning ADG values obtained with the 85CPI diets compared to the 70CPI diets were attributed to the greater starter intake of calves fed 85CPI compared to calves fed 70CPI, which is consistent with a previous report^27^. In addition, many studies show that extensive grain processing maximizes grain digestibility but increases the risk of digestive and health disorders, which negatively affects animal production performance^28,29^. In our experiment, rumen pH was higher in 85CPI calves than in 70CPI calves. However, the forage source had no effect on calf weight gain, which is consistent with our previous results^26^, where ADG was similar between two forage sources, namely WS and AH. The lack of an effect of forage source on intake of starter feeds and ME may be the reason for the similar weight gain between AH and WS in the current study. However, a meta-analysis found that changes in forage sources had a similar effect on ADG after weaning^10^.

Contrary to our hypothesis, no interaction between CPI and forage source was observed in any of the measured performance responses. There could be two explanations for this. First, the proportion of forages was not high enough, especially for AH, to interact with PI. Second, because corn starch has low degradability in the rumen, the claves may not have benefited from forage intake in their diet. In another study, Mirzaei et al.^2^ observed no interaction between physical forms of starter (finely ground vs. textured) and forage supply (corn silage) on FE in dairy calves during the pre and postweaning periods.

In our experiment, calves fed 85CPI had higher FE than those fed 70CPI. Decreasing flake density of grain promotes gelatinization of grain starch, making it more available for microbial digestion in the rumen, resulting in less starch entering the small intestine in beef steers fed high-concentrate diets^13^. On the other hand, an increase in starch fermentation in the rumen may lead to metabolic disturbances and a decrease in DMI^30^. In addition, energy production from rumen fermentation is less efficient than that from intestinal starch digestion and direct glucose absorption^30^. Therefore, improving small intestinal digestion of starch in cattle may improve their production performance. Thus, compared with the 70CPI starter diet, the 85CPI starter diet may shift more starch to the small intestine for digestion (reflected in part by higher rumen pH in 85CPI calves compared with 70CPI calves) rather than being fermented in the rumen.

In the current study, the 85CPI starter feed improved the BW of dairy calves at weaning and at the end of the study. These results do not agree with those of Lesmeister and Heinrichs^16^ who reported that calves fed steam-flaked corn as a starter feed had the lowest ADG in the postweaning period, gained less BW than calves fed dry-rolled corn, and tended to gain less than calves fed whole corn. The discrepancy between our results and other reports may be due in part to differences in CPI, forage source and amount, and mean particle size of steam-flaked corn starters.

### Blood metabolites and rumen pH

In previous studies, calves fed a textured starter exhibited an average rumen pH ranging between 6.10 and 6.30^26^. Notably, our findings during the preweaning phase presented a lower average pH, despite the acknowledged broad variability in mean ruminal pH observed among dairy calves^31^. Our results suggest that the 70CPI starter diets fermented more rapidly in the rumen than 85CPI, which could further lower the rumen pH of calves fed before weaning. Available information suggests that processing of grains and ingredients of starter feeds may alter rumen fermentation patterns and nutrient digestibility in dairy calves^6,32,33^. In this study, calves fed a combination of 70CPI and AH had the lowest pH levels, while those fed 85CPI and WS had the highest levels. Despite similarities in NDF and particle size distribution between the two forage diets, unexpected pH variations occurred. Castells et al.^34^ investigated the effects of different forages on performance and behavior of Holstein calves. Their results showed that calves fed barley straw ruminated longer than calves fed AH, mainly due to the higher uNDF content. This might elucidate the enhanced rumen pH in calves receiving 70CPI starters supplemented with WS. In addition, Ran et al.^12^, found that rumination duration was lower at pH < 5.8 and < 5.6 and that total rumination and chewing time was greater in calves fed a high uNDF diet (by adding barley straw) than in calves fed a low uNDF diet, regardless of barley processing index (65, 75, and 85%). They concluded that increasing the dietary uNDF concentration stimulated rumination when barley was processed with a PI of 65%; therefore, this may be considered an effective strategy to improve rumen pH in heifers fed high grain diets. Our results suggest that the feeding of WS, which has a higher uNDF content than AH, more effectively increased rumen pH in calves fed a 70CPI starter diet compared with AH. However, it should be noted that pH results were based on a single measurement rather than repeated measurements to illustrate pH profiles, which was a limitation of this study.

Similar plasma glucose levels among the treatments in this study indicated that calves were similar in energy status across the diets^6,35^. In the current study, no differences were observed in plasma concentrations of triglycerides and BHB in post-weaned calves. However, plasma BHB concentration was higher in calves fed 85CPI starter diets than those fed 70CPI on d 30. Studies indicated that the increase in the concentration of plasma BHB in preweaning dairy calves is mainly due to the rise in solid feed intake and consequent rumen development^36,37^. Data showed that steam flaking of corn with gelatinization of starch improves the development of the ruminal epithelium^7^ through increased rumen concentrations of volatile fatty acids specifically butyrate, and optimizes ruminal and post-ruminal digestion in dairy calves^8^. Recently, Suarez-Mena et al.^38^ observed a positive linear relationship between starter intake and blood BHB level; however, blood BHB concentration was not only affected by starter feed intake, but it was also affected by the time of day, stress, and intake restriction^6^.

## Conclusions

In our recent study, we hypothesized that the effects of rumen fermentability of carbohydrates—achieved by the degree of corn processing—on the production performance of dairy calves would vary depending on the type of forage the calves received as starter feed. However, the results did not support this assumption. Specifically, no discernible interaction was found between the CPI in the starter feed and the forage source in terms of starter intake, DMI, MEI, FE, and BW. Based on our results, a PI of 85% for corn appears to be optimal for promoting calf growth performance.

## Data Availability

The datasets used and/or analyzed during the current study are available from the corresponding author on reasonable request.
